# Supplementary figures and images for: The mechanism of mammalian proton-coupled peptide transporters
Source: eLife. 2024 Jul 23;13:RP96507. doi: 10.7554/eLife.96507 (PMC11265797; doi:10.7554/eLife.96507)

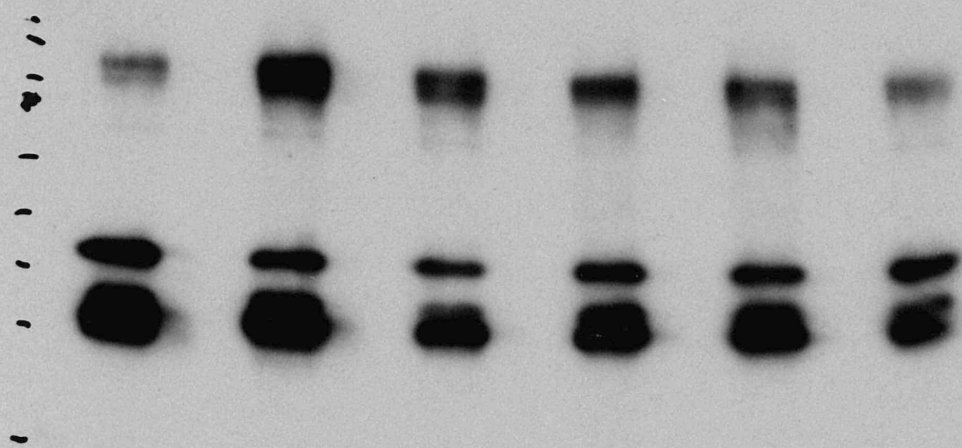

Supplement: Figure 7—source data 1. [file elife-96507-fig7-data1.pdf]

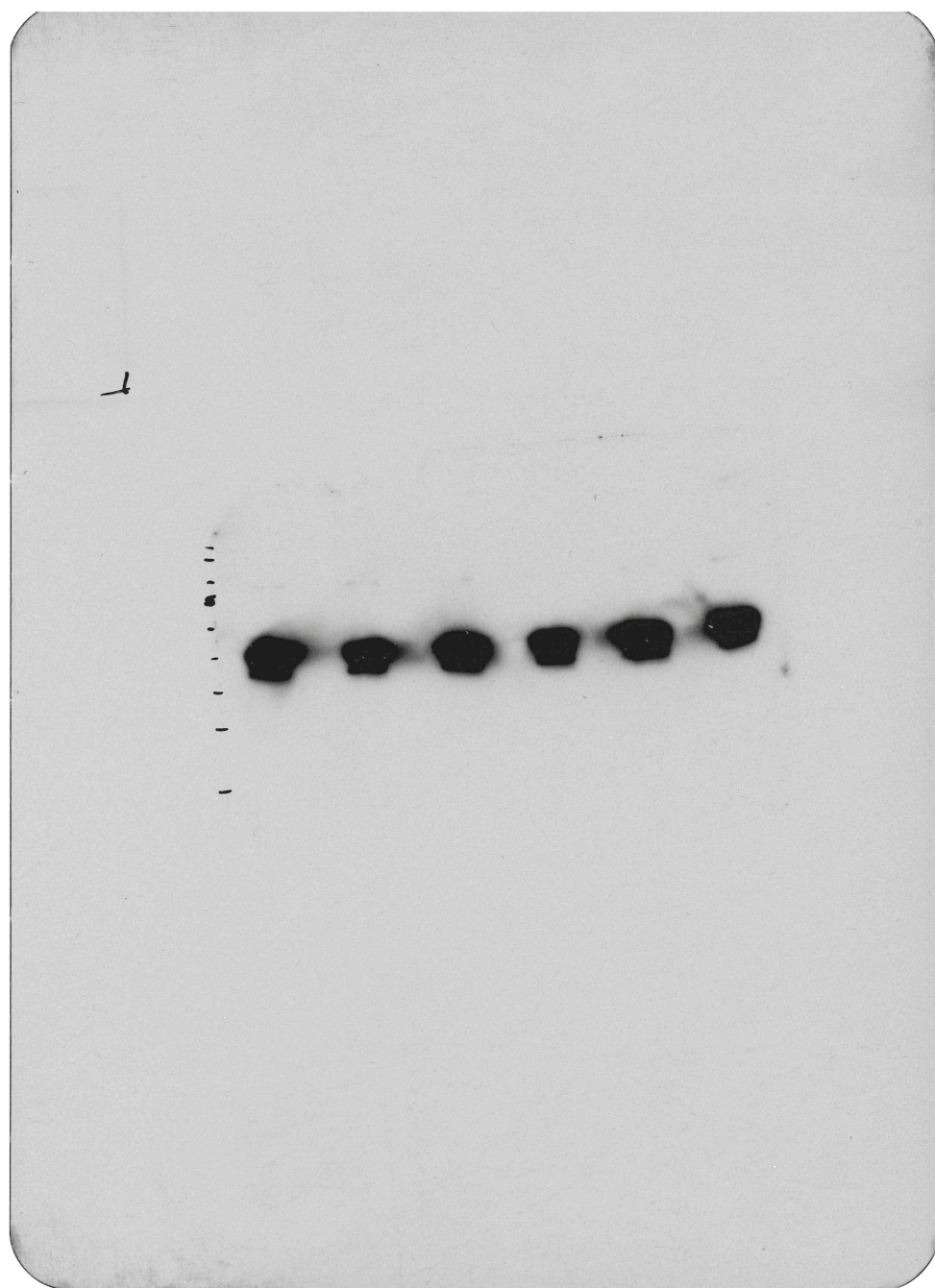

Supplement: Figure 7—figure supplement 1—source data 1. [file elife-96507-fig7-figsupp1-data1.pdf]
